# Phytoecdysteroids: Distribution, Structural Diversity, Biosynthesis, Activity, and Crosstalk with Phytohormones

**DOI:** 10.3390/ijms23158664

**Published:** 2022-08-04

**Authors:** Yamshi Arif, Priyanka Singh, Andrzej Bajguz, Shamsul Hayat

**Affiliations:** 1 Plant Physiology Section, Department of Botany, Faculty of Life Sciences, Aligarh Muslim University, Aligarh 202002, India; 2 Department of Biology and Plant Ecology, Faculty of Biology, University of Bialystok, Ciolkowskiego 1J, 15-245 Bialystok, Poland

**Keywords:** allelopathy, anti-stress activity, isolation, germination, growth regulators, heavy metals, pathogen, salinity

## Abstract

Phytoecdysteroids (PEs) are naturally occurring polyhydroxylated compounds with a structure similar to that of insect molting hormone and the plant hormone brassinosteroids. PEs have a four-ringed skeleton composed of 27, 28, 29, or 30 carbon atoms (derived from plant sterols). The carbon skeleton of ecdysteroid is known as cyclopentanoperhydrophenanthrene and has a β-sidechain on C-17. Plants produce PEs via the mevalonate pathway with the help of the precursor acetyl-CoA. PEs are found in algae, fungi, ferns, gymnosperms, and angiosperms; more than 500 different PEs are found in over 100 terrestrial plants. 20-hydroxyecdysone is the most common PE. PEs exhibit versatile biological roles in plants, invertebrates, and mammals. These compounds contribute to mitigating biotic and abiotic stresses. In plants, PEs play a potent role in enhancing tolerance against insects and nematodes via their allelochemical activity, which increases plant biological and metabolic responses. PEs promote enzymatic and non-enzymatic antioxidant defense systems, which decrease reactive oxygen species in the form of superoxide radicals and hydroxyl radicals and reduce malondialdehyde content. PEs also induce protein biosynthesis and modulate carbohydrate and lipid synthesis. In humans, PEs display biological, pharmacological, and medicinal properties, such as anti-diabetic, antioxidant, anti-microbial, hepatoprotective, hypoglycemic, anti-cancer, anti-inflammatory, antidepressant, and tissue differentiation activity.

## 1. Introduction

**Figure 1:**
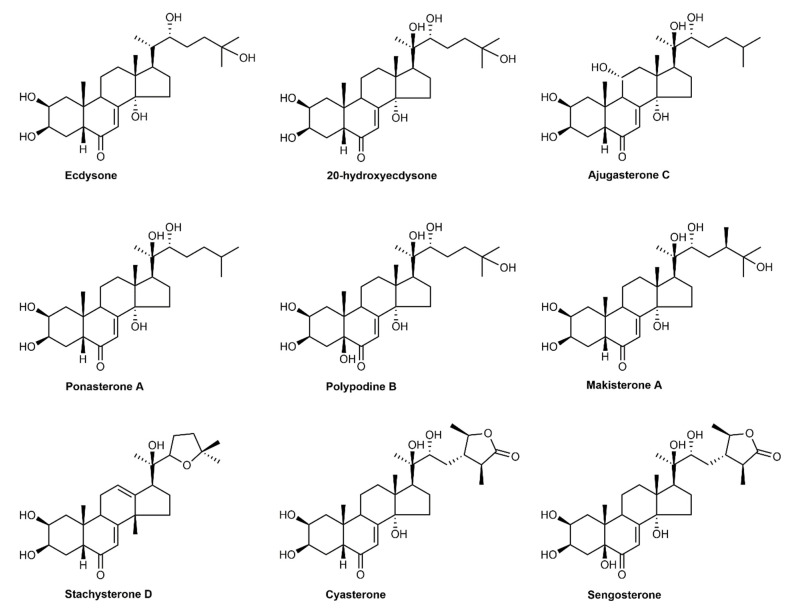
Structures of widely distributed phytoecdysteroids.

The name ecdysteroids (ECs) originates from the Ancient Greek word *ecdysis*, which means “stripping”, ‘‘the shedding of an exoskeleton in insects”. Butenandt and Karlson [1] isolated the first EC—ecdysone—from silkworm pupae. Later, its structure was reported via X-ray crystallography [2]. ECs are steroidal hormones initially found in animals that control insect molting or ecdysis and other important metamorphotic processes in arthropods [3]. ECs have a polyhydroxylated four-ringed skeleton bearing 27–30 carbon atoms derived from cholesterol or other sterols. Depending on the natural source, ECs are subdivided into three groups: phytoecdysteroids (PEs), zooecdysteroids, and mycoecdysteroids. The naturally occurring ECs found in plants are differentiated from the ECs found in animals. However, 20-hydroxyecdysone (20-HE) is the most common and widely used [4,5,6,7]. Many ECs are present in plants and animals, such as 20-HE, ecdysone, ajugasterone C, polypodine B, and cyasterone (Figure 1).

Additionally, PEs are found in algae, fungi, ferns, gymnosperms, and angiosperms. More than 500 different PEs are found in over 100 terrestrial plants. PE distributions in plants vary from organ to organ and may change according to season or geographical area. Some plant species have the genetic capacity to synthesize PEs. However, due to gene transcription suppression in some plant species, PEs are not easily detected due to the limitation of techniques and instrumentation. PE occurrence in plants may be associated with their phylogenetic position. PEs can now be isolated and identified via various chromatographic techniques and nuclear magnetic resonance (NMR) [8,9,10,11].

Arthropod ECs are called zooecdysteroids, and they regulate the growth, development, and other biological functions of arthropods and other invertebrates [12]. In mammals, PEs exhibit versatile growth and metabolic activity; they also display modulatory effects on anabolic and growth traits [13]. PEs isolated from *Serratula coronate* increase ducklings’ vitality, growth, and productivity; and athletes and sportspersons use PEs in low doses for enhanced stimulatory effect on growth and physiological processes [14]. PEs enhance protein synthesis in patients with cancer or acquired immunodeficiency syndrome. PEs also show antidepressant bioactivity, protect the body from stress, and stimulate physical and sexual behavior [15]. In the United States, dietary supplements containing PEs isolated from *Sphenocentrum jollyanum* display antacid and urease inhibitory activities, which have significant effects when treating ulcerative colitis [16]. PEs have a vast pharmacological and medicinal role. PEs display anabolic, adaptogenic, anti-diabetic, hypolipidemic, and hepatoprotective activities. PEs also show anti-microbial, anti-cancer, anti-inflammatory, tissue differentiation, and metabolism-modulator activities in humans. PEs bind and interact with human nuclear receptors. Additionally, PEs also display antioxidant activity that is used to cure several chronic diseases [8,15,17]. In plants, PEs effectively induce biological activities and provide defense against insects, nematodes, and several environmental stresses. They also possess allelochemical activity that provides tolerance against several insect attacks, such as taste receptors, which facilitate hormonal disruptions and toxicity in insects and nematodes. Furthermore, these allelochemicals can alter gene expression profiles in plants via DNA binding [18,19].

This review highlights the latest knowledge on PE distribution, chemistry, isolation, identification, biosynthesis, and regulation. The recent identification of 207 new PEs in 17 plant families is presented. We also highlight chemical transformations and stereochemistry of ECs on the synthesis of EC analogs via esterification, oxidation, reduction, and alkylation. However, complete records on their biosynthesis, distribution, regulation, and role in mammals and plants are scarce, demanding a summary and compilation of present knowledge of PEs. We critically analyze the role of PEs with regard to their biological, pharmacological, and medicinal properties to understand the impact of these phytoconstituents on health and disease. Furthermore, the physio–biochemical roles of PEs in plants and their defensive role against insects, nematodes, fungi, heavy metals, and salinity are highlighted. Additionally, we discuss crosstalk between PEs and phytohormones such as auxins, cytokinins (CKs), gibberellins (GAs), brassinosteroids (BRs), jasmonic acid (JA), and ethylene (ET).

## 2. Distribution of Phytoecdysteroids

Ecdysteroids or related compounds are present in angiosperms, gymnosperms, algae, fungi, aquatic organisms, and some arthropods and invertebrates. The distribution of PEs in plants varies from organ to organ and may change according to season or geographical area. In annual plants, PEs are primarily found in organs such as young tissue, leaves, flowers, anthers, and seeds, whereas they are less frequently present in roots and stems. Accordingly, this may suggest that the highest concentrations of PEs are present in tissues and organs that play an important role in plant growth, development, and survival, or in reproductive organs [8]. However, PEs play a potent role in enhancing plant immunity against predators [18]. In *Chenopodium album*, PE concentrations are the highest in anthers, young leaves, and seeds [20]. In spinach, cotyledons and the first true leaves contain more PEs than the later leaves [21,22].

In annual plants, PEs are translocated from seeds to developing organs such as the shoots and other growing regions. In several perennial plants, PEs seasonal cycling takes place between underground and aerial parts. In particular, during the spring season, the highest concentration of PEs is present in newly developing parts such as shoots. In autumn, PEs are mobilized from the shoot to underground parts, increasing PE levels in the roots [18]. PEs are generally found in a free-state or in conjugated or bound forms with organic acids in ester form (such as benzoate, acetate, crotonate, coumarate, and cinnamate), with sugars in the form of glucosides (such as galactose, glucose, and xylose), with sulfates, or in a methyl ether form or isopropylidene. Additionally, there are some variations of the hydroxyl and conjugating group (such as position, number, and orientation) in the steroid ring structure; sometimes, an oxo group occurs in C-2, C-6, C-12, C-17, C-20, or C-22. These crucial modifications synthesize diverse forms of metabolites in different plants [7].

Zoo- and mycoecdysteroids are present in extremely low concentrations in organisms and cannot be isolated for practical purposes. PEs are found in more than 100 terrestrial fern, gymnosperm, and angiosperm plant families [4,7,18,23,24]. The occurrence of three ECs, named ponasterones A, B, and C, in the leaves of *Podocarpus nakaii* was reported for the first time in plants [25,26]. Simultaneously, 20-HE was isolated from *Podocarpus elatus* wood [27]. These reports have led to further investigations of PEs. ECs are found in more than 50% of fern families, such as Pteridaceae, Polypodiaceae, and Blechneaceae, but are only found in several conifer and angiosperm families [28,29]. Genus *Silene* has large amounts of diverse ECs, i.e., ca. 1–2% of the plant’s dry weight [30]. In *Serratula coronata* leaves and *Leuzea carthamoides* roots, 20-HE content reaches 1.5% (relative to the dry weight of the plant). A high concentration of ECs (4–5%) is found in medicinal plants such as *Achyranthes* and *Cyathula*, which are used in Chinese medicine [28]. However, several plants can synthesize ECs, but the genes responsible are silenced, which may favor plants and pollinators. Spinach and quinoa are the most-edible species that synthesize PEs. The taxons that are used in traditional medicines such as *Leuzea carthamoides*, *Ajuga turkestanica*, and several *Pfaffia* species have abundant ECs in their roots. *Cyanotis vaga* and *Cyanotis arachnoidea* belong to monocot species, and several species of ferns that belong to *Polypodium* are rich sources of ECs [7,8,30,31,32,33,34,35].

About 200 PEs are found in different plant genera, such as *Polypodium*, *Ajuga*, *Silene*, and *Podocarpus* [7,8]. A total of 207 new PEs have been identified from 17 plant families: Amaranthaceae, Asteraceae, Blechnaceae, Caryophyllaceae, Commelinaceae, Dioscoreaceae, Gleicheniaceae, Lamiaceae, Liliaceae, Limnanthaceae, Lygodiaceae, Malvaceae, Menispermaceae, Polypodiaceae, Polyporaceae, Rhodomelaceace, and Taxaceae (Table 1).

## 3. Structure of Phytoecdysteroids

The carbon skeleton of ECs is known as cyclopentanoperhydrophenanthrene, and it contains a β-sidechain at carbon-17. The important features of ECs are the presence of the *cis*-(5β-H) junction of rings A and B, the 7-en-6-one chromophore, and the *trans*-(14α-OH) junction of rings C and D. The sterol structure modulates and synthesizes ECs; subsequently, the *trans*-A/B ring in sterols undergoes conversion into the junction of the *cis*-A/B ring in ECs. These structures are C_27_, C_28_, C_29_, or C_30_ chemical polyhydroxy steroids that bear 14α-hydroxy-7-en-6-one chromophore and the A/B-*cis* ring. 20-hydroxyecdysteroids have been identified for the first time in arthropods, in which they are the main bioactive ECs [8].

ECs are polar steroids, and their solubility is identical to that of sugar molecules; thus, they are lipophilic and soluble in aqueous mediums. However, mammalian steroidal hormones are relatively non-polar and have variable structures. For example, they do not contain polyhydroxylated side-chain features. Additionally, invertebrates cannot synthesize ECs; rather, they consume phytosterols and convert them into ECs. On the other hand, plants produce ECs via mevalonic acid (MVA) and cholesterol [21,22,115,116].

**Figure 2:**
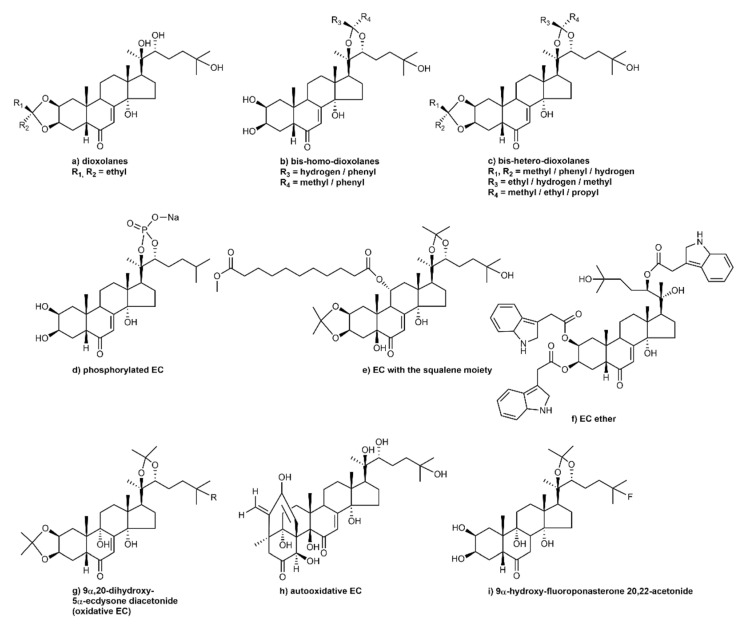

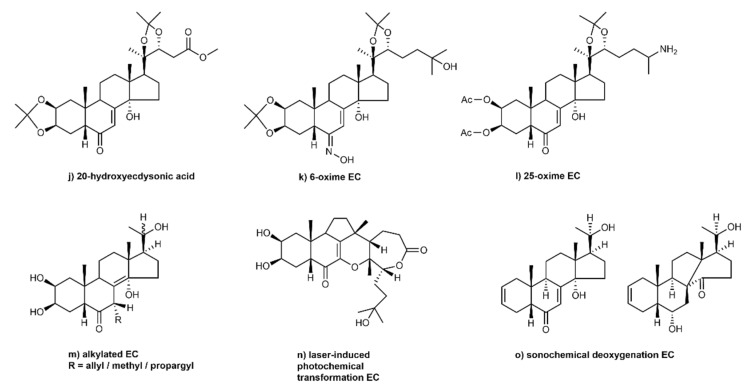
Structural modifications of ccdysteroids via etherification, esterification, oxidation, amination, fluorination, and alkylation.

PEs are found in free-state or conjugated form with sugars (e.g., xylose, glucose, and galactose) as glycosides or with organic acids as esters (such as acetate, cinnamate, benzoate, crotonate, and *p*-coumarate), sulfates, or isopropylidene. Steroid ring structure shows variation, which is not significant; substantial variations are found in the number, positioning, and orientation of hydroxyl groups and conjugating groups. In some cases, the oxo group may be located at different carbon positions, such as C-2, C-12, C-17, C-20, or C-22, along with the required C-6 position. Several structural modifications are present in different plant families, probably due to different uses of metabolites [4,8,15,32]. EC synthesis, stereochemistry, and transformation via etherification, esterification, oxidation, reduction, alkylation, amination, and fluorination (Figure 2) are discussed below.

### 3.1. Etherification of Ecdysteroids

2,3-mono- and 2,3:20,22-bis-dioxolane are new 20-HE derivatives; they are prepared by aldehyde and ketone acid-catalyzed condensation (Figure 2a,b) [28]. When EC is dissolved in methanol (MeOH) prior to the addition of a carbonyl component, new polar EC compounds are formed: 2,3-dioxolane, 20,22-dioxolanes, 2,3:20,22-bis-homodioxolanes, and 2,3:20,22-bis-heterodioxolanes [117]. A similar standard procedure is used for the preparation of 20,22-dioxolanes, bis-homo-dioxolanes, and bis-hetero-dioxolanes (Figure 2a–c). It has been observed that reaction selectivity to cyclic acetal is because of the higher reactivity of the 20,22-diol moiety in contrast to the 2,3-diol moiety. Moreover, in the synthesis of heterodioxolanes, it was observed that the 2,3-dioxolane ring is not synthesized if bulky molecules with benzene-substituted rings are used as reagents [118]. NMR spectroscopy is used to obtain several 20-HE products, including isolated and characterized diastereomers [117].To extend the opportunity for the production of low-polarity ECs and to investigate their chemosensitizing characteristics, poststerone 2,3-dioxolanes have been produced. It has been reported that the treatment of poststerone with methyl isobutyl ketone has two pathways, giving two epimers in equal proportions [119]. It has been observed that in the formation of the stereogenic center at the C-22 position, the bulkiest substituent was located at the β-position. The acyl derivative is a byproduct of the preparation of dioxolane. Therefore, acid-catalyzed condensation is used for the synthesis of nonpolar mono- and bis-dioxolanes of 20-HE and poststerone [28].

A new water-soluble phosphorylated EC analog with a high glycemic index has been synthesized from 20-HE [120]. The synthesis strategy was based on the selective protection of 2,3,20,22-vicinal diol groups and the tertiary 25-OH group. The alkaline oxidation of boronate with hydrogen peroxide and phosphorylation with phosphorous oxychloride in pyridine form 20-HE 20,22-phosphoric acid (Figure 2d) with an overall yield of 67% [28]. The structure–activity relationship showed a large number of semisynthetic EC analogs with the presence of a 2,3-acetonide group, which has chemosensitizing properties against both multi-drug resistant and drug-susceptible cancer cell lines [121]. Moreover, the latest approach to develop nanoscale materials based on EC–squalene conjugates has been reported (Figure 2e) [122]. Many new EC ethers and esters have been synthesized via alkylation and condensation of naturally occurring EC and with the help of several aldehydes and ketones for testing their inhibitory activity against transmembrane protein ABCB1. A large library of new EC ethers and esters has been synthesized by *O*-alkylation or condensation of natural ECs with various aldehydes and ketones for testing of their ability to inhibit the ABCB1 transmembrane protein in vitro (Figure 2f) [123].

### 3.2. Oxidation of Ecdysteroids

The autooxidation of ECs under alkaline conditions was reported several years ago [28]. It has been reported that 20-hydroxyecdysone and ponasterone A diacetonides in 10% methanol (MeOH) solution of sodium hydroxide (NaOH) at room temperature for 3 h via column chromatography resulted in 9α-hydroxy-5α-derivatives isolation, i.e., 9α,20-dihydroxy-5α-ecdysone diacetonide and 9α-hydroxy-5α-ponasterone A diacetonide [124]. Moreover, when NaOH is replaced by potassium carbonate, then it is changed into a 85:15 mixture of initial compounds and its 5α-epimer, which are not easily separated. Furthermore, when the mixture is treated with a 10% MeOH solution of NaOH, it is completely converted into 9α,20-dihydroxy-5α-ecdysone diacetonide. Thus, it can be concluded that hydroxylation takes place after 5β-EC epimerization into the 5α-epimer. Hydrolysis of compounds 9α,20-dihydroxy-5α-ecdysone diacetonide (Figure 2g) and 9α-hydroxy-5α-ponasterone A diacetonide in 10% perchloric acid gives 20,22-monoacetonides and deprotected ECs. One- and two-dimensional NMR spectroscopy are used to determine 9α-OH and 5α-H configurations. Alkaline autooxidation of 5β-ECs continues as stereoselective oxidation and produces its 5α, 9α-hydroxy analogs [28]. Autooxidation of 20-HE with alkaline MeOH solution gives *abeo*-steroid, which has a skeletal rearrangement of 20-HE with cleavage of the bond between the C-3 and C-4 atoms (Figure 2h) [125]. EC oxidation with an ozone or oxygen mixture via pyridine continues chemo- and stereoselectivity to produce natural 2-dehydro-3-epi-20-HE, which has been isolated from the fly *Calliphora erythrocephala*. Furthermore, its hydride reduction yields a diastereomeric mixture of 2α,3α-alcohols and their 2β,3β-analogs. EC 25-fluoroponasterone A diacetonide reaction with lithium in liquid ammonia gives 5β type 9α-hydroxy-EC, which then gives 9α-hydroxy-fluoroponasterone 20,22-acetonide (Figure 2i) [28].

The oxidation of aldehyde with ozone in pyridine and diazomethane solution in diethyl ether synthesizes 23-methoxycarbonyl-25,26,27-tris-nor-20-hydroxyecdysone, which is an analog of 20-hydroxyecdysonic acid (Figure 2j) [28]. Phenyliodine(III) diacetate (PIDA) is used for the oxidative cleavage of EC C20-C22 bonds [126]. Use of PIDA induces production of poststerone from 20E with a yield of up to 81.41%, whereas iodobenzene *I*,*I*-bis(trifluoroacetate) (PIFA) used for this reaction yields only 57.8% [119]. Use of 2-deoxy-20-HE, polypodine B, ajugasterone C, calonysterone, and PIDA reagent provides a higher amount of C-21 E and a lower quantity of byproducts than using PIFA [126]. It has been reported that PIFA acts as a more aggressive reagent due to the release of trifluoroacetic acid, which decreases the reaction chemoselectivity [28].

### 3.3. Oximes of Ecdysteroids

Oximes are used diversely in organic synthesis and display several biological activities. Recently, the synthesis of steroidal oximes has increased. The synthesis of oximes from 20-HE and their later rearrangement into lactams has been studied [127]. Further, several EC oximes are prepared via 20-HE diacetonide using alkoxyamines [128]. It has been found that, depending on the nature of the reaction mixture, this reaction synthesizes either mixtures of ECs (*Z*)- and (*E*)-oximes or their 14,15-anhydrous derivatives; 6(*E*)-oximes are converted to lactam (Figure 2k). The oximation of ketones with hydroxylamine hydrochloride in pyridine and triethylamine (100 °C, 3 h) leads to the formation of several oximes, such as 20- and 25-oximes that had *E*-configuration, identified via X-ray diffraction (Figure 2l) [129].

### 3.4. Alkylation of Ecdysteroids

Alkylation is the major process for steroid-compound modification, e.g., an alkyl group addition at the 7-position. 20-HE treatment with an alkyl halide in lithium ammonia solution forms stereospecific 7α-alkyl 20-hydroxyecdysone derivatives such as 7α-methyl, 7α-ethyl, and 7α-allyl. Allyl derivative synthesis has been used to evaluate the stereospecificity of EC alkylation via X-ray diffraction and NMR. 20-HE reacting with propargyl bromide produces *O*-alkylated, 7α-monoalkylated, and 7,7-dialkylated products. When the amount of propargyl bromide increases, *C*-alkylation products are produced: 7,7-bis(2-propyl-1-yl)-14-deoxy-Δ8(14)-20-HE and *O*-monoalkylation ethers. When the methylation reaction occurs with poststerone, having carbonyl groups at the C-6 and C-20 atoms, the formation of stereoselective 7α-alkyl derivatives takes place, with the reduction of the 20-oxo group producing an equimolar mixture of 20*R*- and 20*S*-hydroxy derivatives; separated by HPLC, these have 94% total yield (Figure 2m). Reaction of excess halide with poststerone gives a diastereomeric mixture of 20-hydroxy-7,7-bis-alkyl EC derivatives. Therefore, alkylation of ECs can lead to a few medicinally potent compounds [130,131,132].

### 3.5. Skeletal Transformations

Few studies have shown that ECs undergo skeletal transformations. Irradiation with UV rays on 20-HE (aqueous solution) synthesized *abeo*-EC [133]. Similarly, the use of laser radiation also caused phototransformation of ECs [134]. Laser irradiation of 20-HE and its diacetonide at 226 nm caused the synthesis of complex mixtures (Figure 2n) and photochemical transformation; apart from synthesizing poststerone, stachysterone B, 14α,15α-epoxy-14,15-dihydrostachysterone B, 14-epi-20-HE, and 14α-hydroperoxy-20-HE, this reaction also gave new products such as lactone, 6-carbaldehyde, and tetrahydroxepine ring-containing skeletal rearrangement products [135]. Ultrasonic reactions have recently been used for the transformation of steroids (Figure 2o). These treatments may influence the conversion process, reaction chemoselectivity, product purity, and yield; further, they may reduce reaction time, inhibit byproduct formation, and reduce catalyst presence [136].

It has been found that sonochemical deoxygenation of poststerone 2,3-dimesylate gives products such as *abeo*-steroid and target products. Treatment using ultrasound rays on poststerone 2,3-dimesylate with a sodium iodide-zinc-dimethylformamide reagent yields 3-dideoxy-∆2(3)-poststerone and/or its (8*R*)-13(14 → 8)-*abeo*-isomer, which are formed by an intramolecular rearrangement. Reaction of *abeo*-pregnanes with complex metal hydrides has been reported for 6- and 20-oxo-groups; however, the 14-oxo-group reactivity was decreased. In 6,20-dioxo-Δ2,3-pregnane and 6,20,14-trioxo-13(14 → 8)-*abeo*-isomer structures, the reduction of hydride is stereospecific, giving 6α,20*R*-diols [137].

## 4. Isolation and Identification of Phytoecdysteroids

Isolation of PEs from plants involves several procedures, including extraction, separation, purification, and identification. The polar nature of PEs makes them difficult to isolate from other major polar plant materials such as chlorophyll, steroids, amino acids, terpenoids, phenols, and pigment constituents. Thus, different chromatographic techniques, such as thin-layer chromatography, normal- and reversed-phase column chromatography, flash chromatography, droplet counter-current chromatography, gel chromatography, and high-performance column chromatography (HPLC) are used for isolation. Rotation planar chromatography (RPC) is an effective preparative method for the separation of ECs that is faster and more effective than preparative TLC [9]. PEs are isolated by solvent extraction of dried plant parts with MeOH or ethanol, followed by a partition with water and hexane. Further, an aqueous portion of the material can be exposed to column chromatography via silica gel, Sephadex^®^ LH-20 (Sigma-Aldrich, Saint Louis, MO, USA), or Diaion^®^ HP-20 (Sigma-Aldrich, Saint Louis, MO, USA). The fraction recovered goes through reverse-phase HPLC using silica gel as the stationary phase. Moreover, normal-phase HPLC, rotation locular countercurrent chromatography, and droplet countercurrent chromatography are also used for PE isolation [138,139]. PEs can be identified using ^13^C- NMR and 2D-NMR; further, they give heteronuclear single quantum coherence or correlation, correlation, heteronuclear multiple bond correlation, rotating frame nuclear Overhauser effect, and nuclear Overhauser effect in spectroscopy. Additionally, carbon resonances of C-2 and C-3, C-14 and C-7, and C-8 lie near δ_C_ 67–69, 83–85, 121–123, and 162–165, respectively. PE chromophores, i.e., 14α-hydroxy-7-en-6-one, can be identified using UV absorption in MeOH at the wavelength (λ_max_) 240–245 nm [8,140].

*Achyranthes bidentata* (Amaranthaceae) contains four furanoECs that have been isolated from the ethanolic extract [141]. The methanolic extracts of *Polypodium vulgare* and *Serratula coronate* roots [28] and *Callisia fragrans* stems [78] have been used for the isolation of PEs. In *Aerva javanica*, the structures of new PEs have been established based on 1D and 2D ^1^H NMR and ^13^C NMR spectroscopy and HREIMS [42]. A new family of zooecdysteroids, called ecdysone lactones, has been isolated from an extract (CH_2_Cl_2_-MeOH, 1:1) of freeze-dried *Antipathozoanthus hickmani*. Ponasterones have been isolated from *Podocarpus nakaii* and *Alcyonidium gelatinosum* via freeze-dried extract (CH_2_Cl_2_-MeOH, 1:1); for their identification, 1D and 2D NMR were applied [28]. Many ECs have been isolated from seeds of *Serratula chinensis* via butanol extract [51].

## 5. Biosynthesis of Phytoecdysteroids

Arthropods and insects cannot cyclize squalene, so they cannot produce sterols; thus, they acquire them from dietary sources. C_27_-sterols are needed for EC biosynthesis, so it has been estimated that several biosynthetic pathways are required in arthropods and insects that permit them to metabolize the C_28_- and C_29_-sterols in plants. However, contrary to plants having a complete biosynthetic pathway for sterols, several plants can produce PEs. In spinach, lathosterol (C_27_-sterol) is the precursor of PEs, whereas in *Drosophila melanogaster*, C_27_-sterol, i.e., cholesterol, synthesizes ECs [4,18,19,23,24,142].

Mevalonic acid (MVA) is the precursor of plant sterol (e.g., triterpenoid) biosynthesis inside the cytosol. Furthermore, isopentenyl diphosphate (IPP) and dimethylallyl diphosphate (DMAPP) are the building blocks of isoprenoid, not only synthesized from MVA but also from the 2C-methyl-erythritol-4-phosphate (MEP) pathway inside plastids. Additionally, in higher plants, IPP and DMAPP from pyruvate and glyceraldehyde-3-phosphate via the non-MVA pathway, and MVA (minor) and MEP (major) pathways are also required for the synthesis of the plant hormone gibberellic acid (GA_3_). In addition, IPP serves as the major component for synthesizing terpenoids. In corn, MVA is not the pathway for synthesizing phytosterols; other pathways participate in synthesizing IPP [18,116,143,144].

The biosynthesis of PEs is based on the cytosolic sterol pathway. Thus, the plant produces PEs mainly from MVA via cholesterol in the mevalonate pathway of the plant cell, using acetyl-CoA as a precursor. In the MVA pathway, six activated isoprene units form squalene in condensation reactions, and this then goes through epoxidation and cyclization to produce lanosterol. In particular, the units eventually go through steps to form cholesterol [13]. Although knowledge of the biosynthesis of PEs is still limited, much study has been done on ECs. The sites of PE biosynthesis have not been found yet, and it is thought to take place in specified cells or tissues. Oxidation of ecdysone to 20-HE takes place via ecdysone 20-monooxygenase; the Δ^7^ sterol has a reduced chain of C-24; this was also shown in *Polypodium vulgare*. However, the precursor of PE biosynthesis is a reduced sidechain at C-24 of Δ^7^ sterol [18].

**Figure 3:**
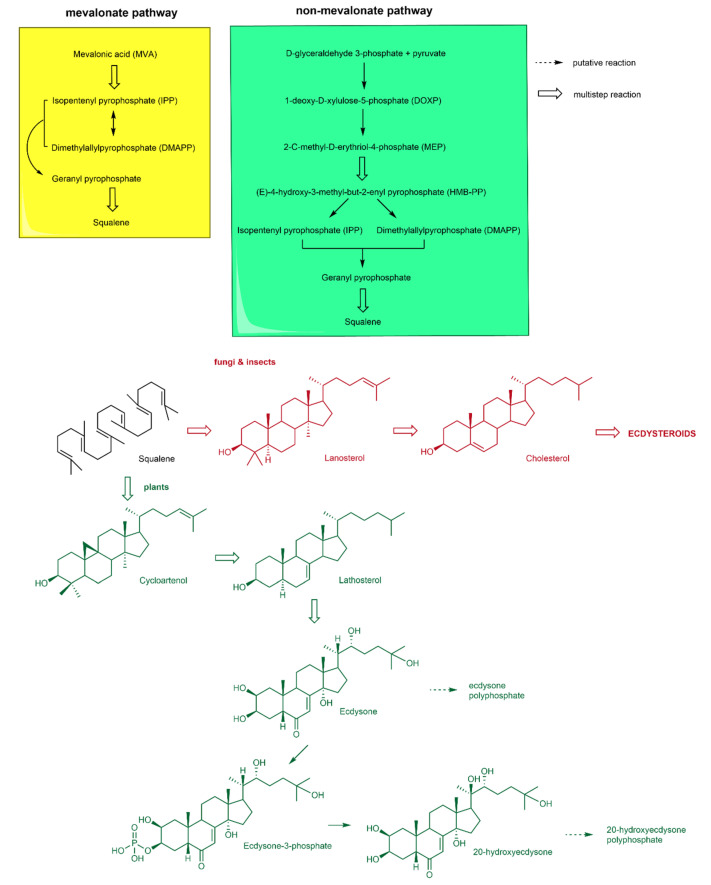
Biosynthesis of phytoecdysteroids.

Furthermore, this is confirmed by using spinach as a model plant for lathosterol, which is Δ^7^ sterol reduced at C-24 [21]. In *Polypodium vulgare* and *Taxas baccata*, a labeled feeding experiment produced labeled E and 20-HE. The location of 20-HE (radiolabeled) biosynthesis is indicated by mobilization of hydrogen from 3α- and 4β-positions to C-4 and C-5 via concomitant 1,2-Wagner–Meerwein hydride transportation from 4β- to 5β- and from 3α- to 4α-positions [8]. Chenopods and other plant families also contain Δ^7^ sterol as PEs precursor. Several plants contain C-24 alkylated sterols, which biosynthesize C-24-alkylated PEs. Furthermore, plants also contain C-24 alkylated sterols and unalkylated cholesterol. Nevertheless, it is still unknown whether lathosterol or cholesterol is the preferred substrate for PE production; both of these sterols are found in *Chenopodium* species [3,4,5,39,142,145]. In *Ajaga reptans* hairy roots, 3β-hydroxy-5β-cholestan-6-one is converted into 2β,3β-dihydroxy-5β-cholestan-6-one and subsequently converted into 20-HE; therefore, this concludes that 7-ene is added afterward during biosynthesis [146]. Figure 3 illustrates the simplified biosynthetic pathway of PEs. Thus, PEs are synthesized from MVA pathways and cholesterol in higher plants; other pathways are still unclear and need to be explored.

### Regulation of Phytzoecdysteroid Biosynthesis

Biosynthetic pathways require strict regulation to control the accumulation of compounds or metabolites. ECs are stable compounds that have slow turnover; thus, ECs accumulate in different organs of the plant and translocate and are distributed from one organ to another [4]. For instance, in spinach (*Spinacia oleracea*), ECs are produced in older leaves (source) and then transported to younger leaves (sink); the removal of the sink or younger leaves leads to the accumulation of ECs in older leaves. Moreover, the biosynthesis of ECs stops in the source. This results in the formation of EC phosphates; this is called the feedback mechanism [4,13]. The same mechanism (no EC phosphates formed) has been reported in *Polypodium vulgare* prothalli, where it actively produces a certain concentration of 20-HE. After applying hot water treatment to prothalli, a complete release of ECs leads to the de novo synthesis of ECs. Notably, this treatment converted 25-hydroxycholesterol into ECs [104,147,148]. In *Taxus* shoots, EC biosynthesis alters with age [4]. PEs provide plants tolerance against several soil nematodes and insects; thus, PE biosynthesis increases during mechanical wounding or pathogenic attack. In *Spinacia oleracea* roots, it has been found that PE production increases during pathogenic attack [21,22,149,150,151].

Meanwhile, it has been demonstrated that JA signaling induces PE and EC biosynthesis during a pathogenic attack [18]. The feedback mechanism inhibits EC biosynthesis in non-accumulating species of plants. For the first time in *Zea mays*, a detectable concentration of ECs was not produced. After long-term labeling, labeled EC and 20-hydroxy EC conjugates were isolated, revealing free EC dispersal after glycosidase treatment. Thus, it has been concluded that every plant synthesizes ECs [152]. Another possible inhibition mechanism was demonstrated in *Allium porrum* (Liliaceae), also known as EC-negative plants [153]. Furthermore, saponins can bind to water-insoluble sterol complexes, therefore abolishing them from existing as EC precursors [154].

## 6. Pharmacological and Bioactivity of Phytoecdysteroids

PEs play a key role in the growth and metabolism of plants; however, they also have several biological and pharmacological activities. They are efficient against several acute and chronic diseases. PEs have several beneficial effects on mammals: they play roles in anabolic, adaptogenic, antidiabetic, anti-inflammatory, antioxidant, antitumor, antimicrobial, and anti-arthritic activity. They also act as hepatoprotectors and immunomodulators [3,8,12,15,155].

PEs have anabolic modulatory activities that are used to treat diabetes, as they have blood-glucose-lowering properties by stimulating β-cells of the pancreas [156]. PEs extracted from the *Ajuga* plant are used to treat diabetes by producing alloxan. In rats, PEs cause a reduction in blood sugar levels; they also decrease urea, nitrogen, cholesterol, lipid peroxidation, and triglycerides in the blood. This further promotes antioxidant enzyme activity, such as catalase, glutathione peroxidase, and superoxide dismutase [157]. PEs increase antioxidant enzyme activity, which reduces glucose oxidation and oxidative stress, which helps to reduce diabetes. Moreover, PEs also have wound-healing and regeneration properties in diabetic animals [155].

The antioxidant properties of PEs are efficient against several chronic diseases. A PE isolated from *Ajuga*, named ajugacetalsterone E, displays cytotoxic activity, generation of superoxide anion, and elastase synthesis in N-formyl-methionyl-leucyl-phenylalanine/cytochalasin B-induced human neutrophils [83]. PEs inhibit and delay collagenase-associated skin damage and oxidative stress [37]. PE derivatives inhibit inflammatory and oxidative enzyme activity, such as thromboxane A2, malondialdehyde (MDA), endothelin, and cyclooxygenase-2, which increases the activity of antioxidant enzymes [158].

Antioxidant activities of PEs have been observed in connection with antimicrobial responses, e.g., 22-epi-ajugasterone C exhibits radical scavenging properties followed by antimicrobial activity. PEs display antimicrobial properties against multi-resistant strains such as *Escherichia coli*, *Staphylococcus aureus*, *Candida albicans*, and *Klebsiella pneumoniae* [60]. PEs also exhibit moderate antibacterial activity against several oral pathogens. Antioxidant and antimicrobial activities are related to anti-inflammatory activity; such bioactivities are observed in several PEs [8]. The PE niuxixinsterone D shows inhibitory effects against lipopolysaccharide-associated nitric oxide synthesis in macrophages. Therefore, it shows anti-neuroinflammatory activity by preventing the production of nitric oxide [141]. PEs such as 3α,14α,22*R*,25-tetrahydroxy-5β(H)-cholest-7-en-6-one possess anti-inflammatory and analgesic activities [75]; α-Ecdysone acts as an immunomodulator and anti-inflammasome [159]. PEs exhibit a potent immunostimulatory effect by increasing the activity of lysosomes and membrane fluidity. Thus, PEs have anti-inflammatory effects in mammals [155].

PEs, e.g., ajugacetalsterone E, inhibit cell proliferation of cell lines as anticancer agents [83]. PEs isolated from *Ajuga forrestii* display cytotoxic activity against human lung cancer cells, hepatoma cells, breast cancer cells, and carcinoma cells [28]. ABCB1 transporters play a key role in chemo-resistance in several tumors and cancer stem cells. PEs exhibit their toxicity against mouse T-cell lymphoma cells and their subcell line transfected with retrovirus overexpressing the ABCB1 efflux pump. PEs inhibit cell proliferation and rhodomine efflux associated with the ABCB1 transporter [121,160]. PEs increase the specific structure–activity relationship for cancer chemotherapy. 20-HE improves antimetastatic and antitumor activity. It was found that mechanistically low doses of PEs and cisplatin affect cells biochemical dynamics, altering DNA and protein biosynthesis in the thymus, liver, pancreas, spleen, and adrenals of tumor-bearing mice. Moreover, high doses of PEs and cisplatin affect metastatic activity [161]. Therefore, PEs act as chemotherapeutic agents with efficient therapeutic effects that modulate cell proliferation and enhance apoptosis, which enhances disease management [28].

PEs display anabolic and adaptogenic effects in mammals, including humans, that activate protein kinase B. Calonysterone has a stronger effect on Akt (a serine/threonine-specific protein kinase) phosphorylation in mammalian skeletal muscle cells [162]. PEs also participate in signal transduction; they act as the downstream effector that modulates protein turnover and skeletal muscle activity. PEs isolated from *Vitex doniana* act as an antidepressant. ECs reduce the adverse effects of stress by inducing the release of glucocorticoids in mice [163]. In quinoa-seed extract, PEs have adaptogenic activity on postmenopausal syndrome [164].

ECs stimulate protein biosynthesis in insects, enhancing growth, gonad development and formation, egg maturation, and protein secretion. ECs and their analogs enhance protein biosynthesis by interacting with insulin signal transduction in insects [165]. PEs also increase the growth and formation of muscle tissue, development of wings, and size of gonads [166]. In warm-blooded species, PEs can regulate fat accumulation and fat-level reduction [167]. They regulate blood sugar levels during alloxan-induced hyperglycemia. They induce glycogen accumulation in the heart and liver. 20-HE promotes liver function after chemical detoxification; it also possesses hepatoprotective properties and induces bile secretion [28]. PEs enhance athletic physical performance, and they are present in dietary products and act as a “natural anabolic agent”. The anabolic effect of ECs enhances physical performance and is mediated by estrogen receptor binding. Thus, this suggests that PEs have several pharmacological uses and bioactivity effects, so they occupy an important position in cosmetics, sports, and military medicine [168].

## 7. Biological Activity of Ecdysteroids in Plants

**Figure 4:**
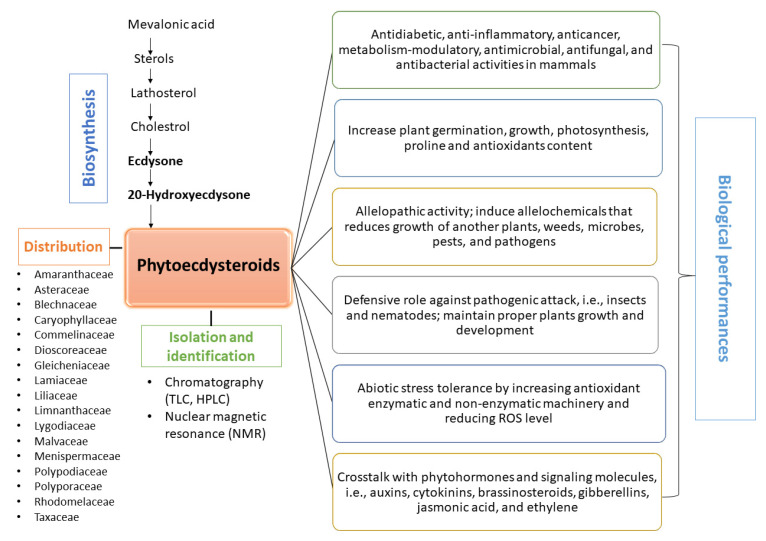
Pictorial representation of distribution, isolation, biosynthesis, and biological roles of phytoecdysteroids.

PEs do not possess hormonal activity in plants; rather, they elicit various biological and metabolic responses (Figure 4). The dry weight of *Leuzea carthamoides* contains many PEs relative to the number of phytohormones [169]. PEs are present in only 2% of plants, and no PE receptors have been reported to date. The EC-binding proteins are nuclear receptor superfamily members, which have a significant domain structure in arthropods. Thus, PEs are secondary plant metabolites and not plant hormones. PEs modulate plant physiological processes and protect against insects and soil nematodes [18].

PEs are crucial for inducing plant physio–biochemical traits and accelerating plant productivity. Germination is crucial in the plant life cycle for further plant growth and development. ECs isolated from *Chenopodium album* have been used to evaluate their bioactivity on *Lactuca sativa* seeds; an aqueous solution of PEs ranging from 10^−4^ to 10^−7^ M was taken. However, the application of PEs did not significantly increase seed germination, root length, or shoot length of *Lactuca sativa* [170]. On the other hand, 20-HE (10^−4^ M) increased germination rate and tomato seedling growth. PEs also exhibit effects such as the ET ‘triple response’ in vegetable seeds [171]. PEs extracted from *Silene viridiflora* have been used to study their effects on different varieties of cotton. Thus, it was found that a 20-HE concentration of 10^−4^ M proved to be more beneficial than 10^−5^ M, as it induced seedling growth in different cotton varieties [172]. Lamhamdi, et al. [173] reported that, in *Triticum aestivum*, pretreatment of 20-HE (3 or 5 µM) efficiently enhanced germination traits, growth parameters, metabolic responses, and antioxidant systems during stress.

In cyanobacterium *Nostoc*, ecdysterone (10^−9^, 10^−7^, or 10^−5^ M) modulates physio–biochemical responses in photosynthesizing living entities and enhances growth [174]. Exogenous ecdysone or 20-HE (10^−10^–10^−8^ M) facilitates the growth and development of the alga *Chlorella vulgaris* [174,175,176,177]. PEs exhibit structural and functional similarities with BRs [19,178]. Exogenous application of 20-HE on *Triticum vulgare* facilitates coleoptile elongation and modulated growth traits [179]. Similarly, PEs (10^−10^–10^−8^ M) promote cell division in unicellular green algae and modulate physio–biochemical and metabolic responses [175,177]. Golovatskaya [179] also found tissue sensitivity towards auxin, displaying a mutual relationship between auxin and PEs. In spinach, auxin, GA, and zeatin significantly affect the accumulation of PEs and further induce plant growth and developmental parameters [151]. PE levels vary during the developmental stages of plants, and a spike in PE concentration is observed during flowering. In *Limnanthes alba*, PE production and accumulation are associated with flowering. The PE level is constant during the growth phase in vegetative tissues; however, it increases drastically during the reproductive stage to increase plant development and protection from predators. Additionally, the seeds of *Limnanthes alba* contain a different form of Pes, which are used extensively in pharmaceutical and medical applications [145]. PEs play a chief role in inducing photosynthesis [19]. In spinach, several PE types are detected in seeds and shoots. Their variation at growth stages is essential for conventional breeding and molecular biotechnology to further promote PE levels in plants, facilitating plant growth and productivity and improving plant immunity against stress [180]. PEs positively regulate photosynthesis in plants. Exogenous application of 2 mM 20-HE on *Tetragonia tetragonioides* increases net photosynthetic rate (P_N_), despite there being no elevation in photosynthetic pigments or electron transport rate. PEs elevate the Calvin cycle (light-independent reactions) and CO_2_ fixation [181]. RuBisCO further catalyzes carboxylation ribulose 1,5-bisphosphate (RuBP) via CO_2_ during the Calvin cycle. Additionally, PE application induces RuBP production [18]. In *Pfaffia glomerata*, the photoperiod enhances the biosynthesis of PEs and further modulates morpho–physiological processes in plants. Additionally, growth, biomass, photosynthesis, and primary and secondary metabolite levels are also enhanced [182]. PEs have several biological activities and potent anabolic activities in plants and animals. Plants such as *Taxus wallichiana*, *Chenopodium quinoa*, *Cupressus tularosa*, *Rhaponticum carthamoides*, *Serratula coronate*, *Datura stramonium*, and *Ajuga* contain the highest amount of PEs in different organs that have several physio–biochemically important functions and/or enhance plant growth and development or boost immunity [183]. PEs are also used in many industries, such as Magnum Thrust from Magnum Nutraceuticals (Surrey, BC, Canada) and Ecdysten from ThermoLife (Phoenix, AZ, USA). Apart from this, the high biological activity of PEs increases the quality and quantity of silk in sericulture industries [184]. *Lychnis flos-cuculi* (ragged robin) contains PEs that provide plants with high medicinal value. Diverse in vitro tissue cultures of this plant, such as micropropagation, shoot cultures, liquid-agitated whole plant cultures with fast-growing roots, and callus culture, produce high amounts of uniform PEs and are regarded as the biotechnological source of biomass rich in pharmaceutically biologically active PEs [185].

## 8. Role of Phytoecdysteroids in Allelopathy

PEs produce allelochemicals, which are chemicals released by plants into the soil that hamper the growth and development of neighboring plants, fungi, microbes, or insects. Extract from roots of *Chenopodium album* containing PEs, such as 20 HE, ecdysone, and polypodine B, has a phytotoxic allelopathic effect on *Lettuce sativa* seeds. It inhibits germination and root and shoot length of lettuce [170]. *Asparagus dumosus* extract containing PEs displays antifungal and antibacterial properties [186]. *Chenopodium quinoa* contains biologically active PEs that have allelopathic potential and suppress germination, growth, and development of weeds and crops [187]. Additionally, quinoa seeds contain many PEs, even more than spinach, with anti-diabetic properties. They decrease blood glucose in obesity [188]. PEs are biologically active allelopathic compounds present in several plants that inhibit the development of neighboring plants, weeds, microbes, and pathogens. They can be used in non-chemical weed management [189]. Thus, there is limited research on the role of PEs in allelopathy, which should be addressed in future research.

## 9. Role of Phytoecdysteroids in Stress

**Figure 5:**
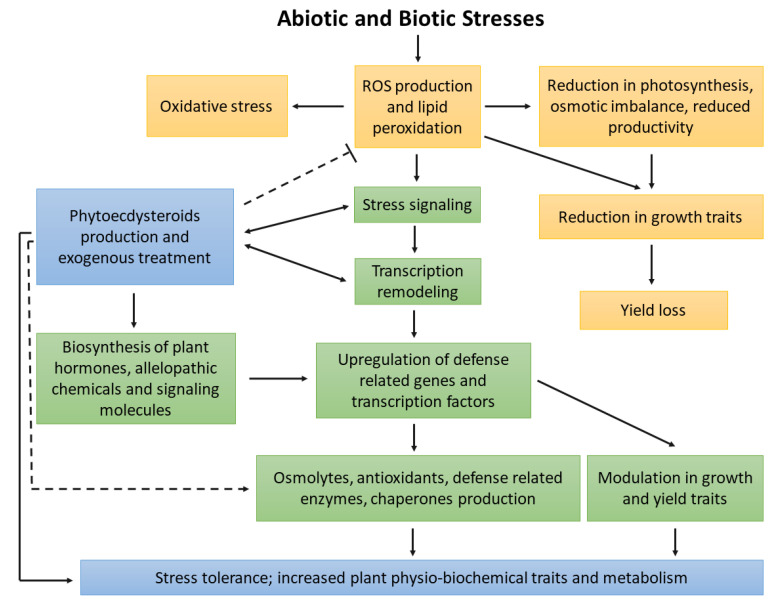
Phytoecdysteroid’s mechanistic approach to enhancing plant stress tolerance.

PEs are polar steroidal secondary metabolites that display a defensive role against biotic (e.g., insects and, nematodes) and abiotic (e.g., heavy metals, radiation, and salinity) stress by increasing the growth, development, and biochemical responses that reduce the lethality of stress (Figure 5).

### 9.1. Biotic Stress

PEs are insect molting hormones that accumulate in several plant species and are regarded as potent allelochemicals that enhance plant tolerance against phytophagous insects and nematodes. PEs exhibit antistress activity in animals, algae, and plants [173]. PEs enhance abnormal molting in several arthropods, having a lethal effect. Further, they reduce mechanical damage, wounding, and insect herbivory. Therefore, inducing internal PE concentrations or alternating PE profiles via breeding and genetic modifications provides essential plant tolerance strategies. PEs alone or in combination with other potent signaling molecules deter plant consumption (antifeedants), disrupt invertebrates’ endocrine system, or cause death in phytophagous insects. This hypothesis can be supported via several experimental results involving PE application on ototransgenic plants to enhance PE levels [190]. For example, the exogenous application of ecdysone in tomatoes reduced root-knot nematode *Meloidogyne incognita* infection and enhanced tomato tolerance [191]. The level of PEs was increased after JA treatment, and the infestation of nematodes (cyst nematodes and root-knot nematodes) was reduced [18]. In spinach, several PEs that protect the plant from insects and induce plant fitness and yield are found in different concentrations at different phases of the plants’ life cycle [180]. Similarly, in *Limnanthes alba*, the PE concentration was highest during the reproductive stage, enhancing plant growth and development and protecting against insect predators [145]. PE treatment negatively affects invertebrate growth, development, and reproduction, but PEs do not exhibit toxicity in mammals [3]. PEs inhibit the feeding of the spring wheat aphid (*Schizaphis graminum*), which causes chlorosis, necrosis, and senescence in wheat. 20-HE and its analog ponasterone A inhibit ecdysis of pink bollworm *Pectinophora gossypiella* [192].

PEs play a crucial role in enhancing immunity against plant-parasitic nematodes. It has been reported that PEs play a protective role in *Spinacia oleracea* against four classes of nematodes: *Heterodera avenae* (cereal cyst nematode), *Heterodera schachtii* (sugarbeet cyst nematode), *Pratylenchus neglectus* (root-lesion nematode), and *Meloidogyne javanica* (root-knot nematode). PEs increase abnormal molting and immobility and decrease invasion and development, leading to nematode death; thus, PEs protect plants against plant-parasitic nematodes and enhance stress tolerance [190].

Spinach roots can be infested with black vine weevil (*Otiorhynchus sulcatus*) larvae, which increases 20-HE concentrations in roots in response to root damage caused by insects. Thus, it can be concluded that wounding increases root 20-HE accumulation as the result of induced de novo 20-HE synthesis in the root [151]. In *Spinacia oleracea*, root damage and root herbivory by larvae of the dark-winged fungus gnat (*Bradysia impatiens*) increases the accumulation of PEs. Induction of PEs along with JA reduces the toxic effect of *Bradysia impatiens* on plants. Therefore, PEs act as an inducible defense and contribute to protection against insect herbivory [193]. It has been found that wounding caused by pathogens increases accumulations of PEs in the roots, with signaling via an octadecanoic acid pathway or endogenous JA, resulting in enhanced resistance against subterranean herbivorous insects (*Spodoptera exigua*) and fungi [150]. In the bracken fern (*Pteridium aquilinum*), PEs play a potent defensive role in enhancing immunity against phytophagous insect attacks [194]. *Meloidogyne* spp. impair plant root development; in *Pfaffia glomerata*, 20-HE displays susceptibility and resistance to *Meloidogyne* infection and histological alteration in roots [34]. In *Achyranthes japonica*, PE accumulation has been reported in reproductive organs such as seeds and roots, indicating that PEs have a protective role against phytophagous insects, especially in reproductive organs or newly developing organs [195]. Based on liquid chromatography–time of flight–mass spectrometry analysis, PEs can be found in large quantities in leaves and roots of genus *Ajuga*; thus, its extract can be used to control phytophagous insect growth in pest-management programs [196].

### 9.2. Abiotic Stress

In spinach, 20-HE plays a potent role in enhancing stress tolerance. It stimulates the activity of antioxidant enzymes, proteins, and proline, mitigating environmental stress and enhancing plant metabolic responses to respond better to any abiotic stress [149]. In *Triticum aestivum*, lead (Pb) stress is alleviated by applying 20-HE; it reduces Pb absorption and alters membrane permeability to Pb. Further, at the intracellular level, 20-HE increases sequestration and chelation of Pb and enhances the accumulation of proline, proteins, metallothioneins, and glutathione. Additionally, 20-HE increases the content of antioxidant enzymes such as superoxide dismutase (SOD), ascorbate peroxidase, and glutathione reductase, which reduce reactive oxygen species (ROS) in the form of superoxide radicals and hydroxyl radicals and decreases malondialdehyde (MDA) content. Thus, it can be concluded that PEs possess antioxidant and radical-scavenging properties, which help enhance plant tolerance against stress [173]. In *Chlorella vulgaris* cells, Pb stress reduces cell growth and development and induces cellular fragmentation and lysis. However, the application of 20-HE restores damage done by Pb stress by increasing growth and chlorophyll and protein content and by decreasing cellular Pb levels [176]. Likewise, γ-irradiation causes abiotic stress and disrupts membrane stability in plants. However, low doses of γ-irradiation on *Sesuvium portulacastrum* have proven feasible and eco-friendly. They further enhance the productivity of 20-HE in vitro in multiple shoot cultures and further induce sustenance. A high-productivity putative mutant that produces a high level of 20-HE has been used to propagate PEs on a large scale for commercial use [184].

Salinity is a major abiotic stress that impairs plant growth and development and hampers sustainable agriculture practices [197]. However, *Pfaffia glomerata* moderates salinity by increasing the production of 20-HE, salicylic acid, osmolytes, and antioxidants. Additionally, salinity upregulates several PE biosynthesis genes and other genes such as *Spook* and *Phantom*, enhancing PE production [198]. In *Gossypium hirsutum* (cotton), application of 20-HE elevates salinity stress and induces germination rate and seedling growth [172]. In spinach, various elicitors, such as methyl salicylate and mechanical damage, facilitate the production of PEs, which remarkably induce metabolic and anabolic activity in plants [199]. PEs alleviate salt stress in wheat seedlings by increasing the activity of antioxidants such as catalase, peroxidase, and SOD and the contents of ascorbic acid and glutathione, which reduce ROS and MDA content and promote wheat tolerance to salinity [200]. In *Silene claviformis*, the sum of PEs exhibits potent stress-protective activity [201]. To our knowledge, no study has addressed the role of PEs in response to other environmental stresses, such as drought, heat, cold, and other heavy metals; this needs to be further explored.

## 10. Phytoecdysteroid Crosstalk with Plant Hormones

This section discusses possible relationships between PEs and plant hormones (i.e., auxins, GAs, CKs, ET, BRs, and jasmonates) to modulate plant-growth developmental processes (Figure 3 and Figure 4).

### 10.1. Auxins

In cell suspension culture from *Serratula tinctoria*, free and esterified PEs have been found, including 20-HE, polypodine B, and 20-HE-3-acetate [202]. The addition of 2,4-dichlorophenoxy acetic acid (2,4-D), which is synthetic auxin in the cultures of a plant transformed by *Agrobacterium rhizogenes*, displays a dose-dependent inhibitory effect on PE concentration. Moreover, the addition of 2,4-D (0.05–0.5 mg/L) reduces hairy root growth, while a higher concentration induces necrosis [203]. In *Ajuga reptans*, phosphate reduction leads to an increase in 20-HE content, while adding indole-3-acetic acid enhances *Ajuga* hairy root growth rate due to induction of the root apical meristem [204]. 20-HE shows the same results as auxin (i.e., naphthalene-3-acetic acid) in tomatoes, with both modulating germination and reducing protein content [171]. In wheat coleoptiles, an assay of PEs with auxin-like activity showed that ECs do not have any biophysiological activity, but a higher concentration of ECs had an inhibitory effect on plant physio–biochemical traits [205]. No published study has addressed any synergistic or antagonistic relationship between auxin and PEs; the impact of exogenous application of ECs on the endogenous level of auxins has not been described and needs to be explored.

### 10.2. Cytokinins

Cytokinins (CKs) are an essential class of plant hormone that modulates plant growth and developmental traits. CKs are adenine-like compounds substituted with isoprenoid or aromatic rings at the *N*^6^ position. They display no structural resemblance to PEs in plants; they are found in plants as free-species glycosides, ribotides, or ribosides. Isopentenyladenosine (iPR) is also present in animals and has anti-cancer properties in mammals, while it enhances plant cellular and metabolic processes in plants [18]. In plants, the callus of dedifferentiated cells can increase and grow to a large extent in a disorganized manner, similar to human cancerous cells; thus, iPR showed pronounced effects in plants in the re-differentiation of cells to form adventitious buds [206]. Furthermore, iPR conjugated with ECs is found in insects; this compound has been identified as 22-*N*^6^-(isopentenyl) adenosine monophosphoric ester of ecdysone using NMR and mass spectroscopy [18]. In *Locusta migratoria* (migratory locust), 22-*N*^6^-(isopentenyl) adenosine monophosphoric ester of ecdysone was found in high concentration in newly laid eggs [207].

Furthermore, a higher concentration of a 2-deoxyecdysone conjugate with adenosine monophosphate has been noted. It has been reported that insects can synthesize 22-phosphate conjugates of 2-deoxy-20-HE, 20-HE acetate, and 20-HE; meanwhile, in female locusts and related species, all these conjugates are hydrolyzed during embryonic development, synthesizing biologically active hormones [208,209]. In *Drosophila*, 6-benzylaminopurine, which is an aromatic CK, enhances growth and development; thus, it can be concluded that CKs play a powerful role in inducing growth and developmental process in insects [210].

Additionally, 6-furfurylaminopurine delays senescence and aging (increased lifespan) in several plants and *Zaprionus paravittiger* (fruit flies). Therefore, the anti-aging bioactivity of CKs reduces age-associated deaths in adults by reducing development during larval and pupal stages [211]. Thus, there is limited research on the role of crosstalk between CKs and PEs in plants.

### 10.3. Brassinosteroids

Brassinosteroids (BRs) show structural resemblance with PEs; they show bioactivity in insects as weak and unstable EC antagonists. BRs and PEs both comprise triterpenoid families, i.e., polyhydroxylated steroids C_27_ and C_29_ with an oxygenated B-ring. Furthermore, the B-ring of BRs and their analogs contains a carbonyl group at C-6, which is further expanded to form lactone, while ECs have a 14α-hydroxy-7-en-6-one group. Moreover, hydroxyl groups located at C-2, C-3, and C-22 are found in both BRs and PEs, but they differ in orientation and location of the hydroxyl group. In ECs, A- and B-ring junctions have *cis*-configuration, whereas in BRs they bear A/B *trans* orientation. Because of such structural variations, BR receptors cannot recognize ECs, and EC receptors cannot recognize BRs; this is the primary reason behind the specificity between plant BR and insect EC receptors. Due to this, PEs and BRs coexist in plants and do not interfere with signaling pathways [15,19,212,213].

ECs display weak or no bioactivity inside the bioassay of BR-responsive plants. BRs do not interrupt EC signaling inside insects due to receptor specificity, and this may have adverse effects because BRs are present in the plants and plant tissues consumed by insects [205]. In *Oryza sativa* lamina inclination bioassay and *Drosophila melanogaster* BII cell bioassay, synthetic analogs of BRs and ECs are made in labs to detect their synergistic or antagonist relationships. Among them, only a single compound shows a PE-synergistic relationship, albeit at higher doses and with weaker activity (2000-fold) than 20-HE [214]. Most of the substances show activity in the BR bioassay; it has been found that biological activity decreases as the structure deviates from that of castasterone, which is used as a standard for BRs. PEs and BRs participate in plant growth and developmental processes by modulating photosynthetic traits. The application of BRs mitigates stress by increasing photosynthetic membrane stability, reorganization of major photosynthetic pigment complexes, and slightly unstacking the thylakoid membrane [215,216]; PEs show similar functions [19,183]. Application of brassinolide (BL) (10^−8^ or 10^−6^ M) alters PE content to a certain degree, which largely depends on leaves’ developmental stages. It has been demonstrated that PE concentration is higher in older leaves of the control set than in plants treated with 10^−6^ M BL, whereas 10^−8^ M BL induced PE content. This effect is weaker and temporary. Further, the exogenous application of BL changes the PE profile in younger leaves. In younger leaves of the control set, a higher concentration of polypodine B is found, whereas in older leaves, lower contents of ajugasterone C and stachysterone are found. However, the application of BRs reduces polypodine B and stachysterone content and enhances ajugasterone C content. Thus, it can be concluded that BR application leads to modulation of PE profiles inside plant tissues within hours [217].

### 10.4. Jasmonic Acid

Jasmonic acid (JA) and its derivatives play a potent role in enhancing plant tolerance against various biotic and abiotic stresses. JA rapidly accumulates during plant mechanical wounding or after an attack by pathogens, e.g., herbivores or insects. Methyl ester of JA (methyl jasmonate, MeJA) enhances JA signaling after a pathogenic attack. However, it also increases the synthesis of defense proteins. Subsequently, MeJA is converted into JA-isoleucine; it is known that these compounds participate in plant stress tolerance. These responses validate the effective role of PEs in boosting plant immunity during a pathogenic attack [218]. In hydroponically grown spinach, increases in PE concentrations have been reported after MeJA application. It has been found that mechanically damaged roots showed a higher accumulation of 20-HE, by about 3-fold, within 2 days. However, after 2 days, induction of PE concentration was identified in shoots. Nevertheless, shoot concentration was unchanged after *Spodoptera exigua* (insect) attack. Thus, it was concluded that endogenous JA in the roots mediates signals during wounding and induces PE accumulation [150,193].

Further experiments on spinach using the same inducible system found that, after an attack by *Bradysia impatiens* (dark-winged gnat fungus), MeJA application increased PE production about four- to seven-fold in roots. Furthermore, 20-HE accumulation reduced larval establishment of dark-winged gnat fungus. Thus, PEs are potently inducible against attacks by insects and herbivores [193]. PEs cause immobility, abnormal molting, decreased invasiveness, reduced development, and death in plant-parasitic nematodes [190]. However, studies must be carried out to assess the synergistic role of JA and PEs during defense signaling.

### 10.5. Other Phytohormones

There is little research on the crosstalk between GAs and PEs. In a dwarf *Zea mays* GA bioassay, treatment with 20-HE and ecdysone, the two insect-molting hormones, do not affect plants; whereas in a dwarf *Oryza sativa* bioassay, a slight GA-like activity is reported [205]. Thus, it can be concluded that PEs show no activity in maize, but in rice, synergistic activity of PEs with GAs is noted. In *Lycopersicum esculentum*, 20-HE and GA_3_ mutually induce germination rate, shoot–root length, and proline content [171]. In *Chenopodium rubrum* and dwarf *Zea mays*, ecdysone application results in slight ET production. Additionally, after ET production and its effect disappear, ecdysone has a tiny effect on ET biosynthesis and bioactivity [18].

To our knowledge, to date, no studies have reported on PE’s relationship with other plant hormones, such as abscisic acid, salicylic acid, and melatonin, which needs to be further explored.

## 11. Conclusions and Future Prospects

PEs are naturally occurring polar steroidal secondary metabolites that have versatile uses in invertebrates, animals, and plants. Invertebrates cannot synthesize ECs, so they consume phytosterols and convert them into ECs; however, plants produce ECs via MVA and cholesterol. PEs can be used as a cheap and sustainable compound that have beneficial effects on both plants and animals. Promising biological and pharmacological activities of various PEs are known, and they may be utilized in the development of nutraceutical and pharmaceutical products after further confirmatory research on their efficacy and safety. Several studies report the biological properties of PEs in animals, such as anti-diabetic, anti-microbial, hepatoprotective, hypoglycemic, anti-cancer, anti-inflammatory, immunomodulatory, and tissue differentiating activity. However, studies addressing the roles of PEs in plants are still scarce. PEs are potently induced against pathogenic attack; they cause immobility, abnormal molting, decreased invasiveness, reduced development, and death in plant pathogens. Additionally, PEs promote antioxidant machinery, reducing ROS and MDA accumulation caused by biotic and several environmental stresses, such as salinity and heavy metals, improving plant growth and biochemistry. PEs crosstalk with plant hormones such as auxins, CKs, GAs, BRs, JA, and ET, which play major roles in developmental growth processes and induce plant stress tolerance.

As the world is facing several kinds of biotic and abiotic stresses that hamper plant growth and development, we need a compound that is cheap and eco-friendly that reduces the global food crisis and can feed every mouth. Therefore, the hypothesis is that PEs are efficient steroid secondary metabolites that majorly modulate growth and developmental processes in healthy and stressed plants. PEs have allelopathic properties that induce biological weed and microbe management and are essential for sustainable agriculture. PEs improve germination, growth, photosynthesis, antioxidant activity, and osmolyte content to enhance plant tolerance against biotic and abiotic stress. Furthermore, unraveling the contribution of PEs in enhancing biotic and abiotic stress tolerance is of great use and needs to be explored. More research should be conducted on PEs’ association with plant hormones. The interaction of PEs with signaling molecules in enhancing stress tolerance needs to be studied. Further analysis of the origins of isoprenoid units utilized in the biosynthesis of PEs is also required. Furthermore, more investigations should be conducted on PEs’ regulation of genes, proteins, and metabolites to modulate plants’ biological performance under normal and stress conditions. Various areas need to be further explored.

## Figures and Tables

**Table 1:** Distribution of phytoecdysteroids in selected plant families.

Family	Species	Phytoecdysteroid	References
Amaranthaceae	*Chenopodium quinoa*	kancollosterone	[36]
		20,26-dihydroxy 28-methyl ecdysone	[37]
		20,26-dihydroxy 24(28)-dehydroecdysone	
		20-hydroxyecdysone 22-glycolate	
	*Chenopodium album*	5β-hydroxy-24(28)-dehydromakisterone A	[38]
		3β,14α-dihydroxy-5β-pregn-7-ene-2,6,20-trione	[39]
		24,25-dehydroinokosterone	
		25,27-dehydroinokosterone	
	*Achyranthes bidentata*	niuxixinsterone A	[40]
		niuxixinsterone B	[41]
		niuxixinsterone C	
		niuxixinsterone D	
		(25*S*)-20,22-*O*-(*R*-ethylidene)inokosterone	
		20,22-*O*-(*R*-3-methoxycarbonyl)propylidene-20-hydroxyecdysone	
		achyranthesterone A	
		(20*R*,22*R*)-2β,3β,20,22,26-pentahydroxy-cholestan-7,12-dien-6-one	
	*Aerva javanica*	aervecdysteroid A	[42]
		aervecdysteroid B	
		aervecdysteroid C	
		aervecdysteroid D	
	*Froelichia floridana*	2,22-dideoxy-20-hydroxyecdysone 25-*O*-β-d-glucopyranoside	[43]
		2,22-dideoxyecdysone 25-*O*-β-d-glucopyranosyl-(1→2)-β-d-glucopyranoside	
		2,22-deoxyecdysone 25-*O*-β-d-glucopyranoside	
		5α-2,22-dideoxyecdysone 25-*O*-β-d-glucopyranosyl-(1→2)-β-d-glucopyranoside	
		2,22-dideoxy-5β-hydroxyecdysone 25-*O*-β-d-glucopyranosyl-(1→2)-β-d-glucopyranoside	
	*Cyathula officinalis*	24-hydroxycyasterone	[44]
		isopropylidene cyasterone	
		2,3-isopropylidene isocyasterone	
	*Atriplex portulacoides*	septanoecdysone	[45]
	*Rhagodia baccata*	(20*R*)-22-deoxy-20,21-dihydroxyecdysone	[46]
	*Pfaffia glomerata*	pfaffiaglycosides C	[47]
		pfaffiaglycosides D	
		pfaffiaglycosides E	
Asteraceae	*Leuzea carthamoides*	rapisterone D 20-acetate	[48]
		inokosterone 20,22-acetonide	[49]
		carthamoleusterone	[50]
		integristerone A 20,22-acetonide	
		15-hydroxyponasterone A	
		14-epi-ponasterone A 22-*O*-β-d-glucopyranoside	
		22-deoxy-28-hydroxymakisterone C	
		26-hydroxymakisterone C	
		1β-hydroxymakisterone C	
		(24*Z*)-29-hydroxy-24(28)-dehydromakisterone C	
		lesterone	
		leuzeasterone	
	*Serratula chinensis*	20-hydroxyecdysone-2-*O*-β-d-galactopyranoside	[51]
		3-*O*-acetyl-20-hydroxyecdysone-2-*O*-β-d-glucopyranoside	[52]
		24-*O*-acetyl-epi-abutasterone	
		3-*O*-acetyl-20-hydroxyecdysone-2-*O*-β-d-galactopyranoside	
		20-hydroxyecdysone-20,22-butylidene acetal	
	*Rhaponticum uniflorum*	rhapontisterone	[53]
		turkesterone 2-*O*-cinnamate	[54]
		makisterone C-20,22-acetonide	[55]
		ajugasterone C-2,3,20,22-diacetonide	
		5-deoxykaladasterone-20,22-monoacetonide	
		uniflorsterone	
	*Serratula coronate*	coronatasterone (2-deoxy-3-epi-4β,20-dihydroxyecdysone)	[56]
		ecdysone 22-acetate	
		ajugasterone 11-acetate	
		3-epi-20-hydroxyecdysone	
		(25*S*)-inokosterone 26-acetate	
		20,22-*O*-(*R*-ethylidene)-20-hydroxyecdysone	
		20,22-*O*-(*R*-ethylidene)-ajugasterone C	
	*Serratula wolffii*	11α-hydroxypoststerone	[57]
		herkesterone	[58]
		25-hydroxydacryhainansterone	[59]
		14-epi-20-hydroxyecdysone	
		2β,3β,20*R*,22*R*,25-pentahydroxy-5β-cholest-6,8(14)-dien	
		24-methylene-shidasterone	
		14α,15α-epoxy-14,15-dihydrostachysterone B	
		20,22-didehydro taxisterone	
		1-hydroxy-20,22-didehydrotaxisterone	
		serfurosterone A	
		serfurosterone B	
		14,15α-epoxy-(20*R*,22*R*)-2β,3β,20,22,25-pentahydroxy-5β-cholesta-7,14-dien-6-one	
		(20*R*,22*R*)-2β,3α,20,22,25-pentahydroxy-5β-cholesta-7-en-6-one	
		22-methylene-2β,3β,11α,14α,25-pentahydroxy-5β-cholesta-7-en-6-one	
		2β,3β,14α,25-tetrahydroxy-5β-cholesta-7,20(22)-dien-6-one	
		1β,2β,3β,14α,25-pentahydroxy-5β-cholesta-7,20(22)-dien-6-one	
	*Serratula cichoracea*	22-epi-ajugasterone C	[60]
	*Serratula strangulate*	(24*R*)-24-(2-hydroxyethyl)-20-hydroxyecdysone	[61]
	*Klaseopsis chinensis*	25,26-didehydroponasterone A	[62]
		stachysterone C	
Blechnaceae	*Brainea insignis*	brainesteroside A	[29]
		brainesteroside B	
		brainesteroside C	
		brainesteroside D	
		brainesteroside E	
Caryophyllaceae	*Silene brahuica*	integristerone A 25-acetate	[63]
	*Silene wallichiana*	2-dehydroxyecdysterone-3-*O*-benzoate	[64]
		2-deoxyecdysterone-25-acetate	
	*Silene viridiflora*	5α-2-deoxy-20-hydroxyecdysone 20,22-acetonide	[65]
		makisterone C	
	*Silene wolffii*	(11α)-11-hydroxyshidasterone	[7]
		(2β,3β,5β,14β,22*R*)-2,3,20,22,25-pentahydroxycholest-7-en-6-one	
		(2β,3α,5β,14α,22*R*)-2,3,20,22,25-pentahydroxycholest-7-en-6-one	
		22-dehydro-20-deoxy ajugasterone C	
		1-hydroxy-22-deoxy-20,21-didehydroecdysone	
		22-deoxy-20,21-didehydro ecdysone	
		ponasterone A-22-apioside	
		3-epi-shidasterone	
	*Silene frivaldszkyana*	26-hydroxyintegristerone A	[11]
	*Silene gigantean*	2-deoxy-20-hydroxyecdysone 25-glucoside	[11]
	*Silene brahuica*	22-*O*-α-d-galactosylintegristerone A 25-acetate (sileneoside H)	[66]
	*Silene italic*	9α,20-dihydroxyecdysone	[67]
		9β,20-dihydroxyecdysone	
		2-deoxy-20-hydroxyecdysone-22-*O*-β-d-glucopyranoside	
	*Silene montbretiana*	3-*O*-β-d-glucopyranosyl-3β,25-dihydroxy-5β-cholest-7-en-6-one-25-*O*-β-d-glucopyranoside	[68]
	*Silene guntensis*	2,3-diacetate-22-benzoate-20-hydroxyecdysone	[69]
	*Silene pseudotites*	2-deoxyecdysone 22β-d-glucoside	[70]
		2-deoxy-20,26-dihydroxyecdysone	
		2-deoxypolypodine B 3β-d-glucoside	
	*Silene otites*	2-deoxy-21-hydroxyecdysone	[71]
		5α-2-deoxy-21-hydroxyecdysone	
	*Silene viridiflora*	2-deoxy-5,20,26-trihydroxy ecdysone	[72]
		5,20,26-trihydroxyecdysone 20,22-acetonide	[73]
		2-deoxy-5,20,26-trihydroxyecdysone 20,22-acetonide	[65]
		20,26-dihydroxyecdysone 20,22-acetonide	[74]
		20-hydroxyecdysone 20,22-monoacetonide-25-acetate	
		2,22-diacetate-20,26-dihydroxyecdysone	
		3,22-diacetate-20,26-dihydroxyecdysone	
	*Acanthophyllum gypsophiloides*	3α,14α,22*R*,25-tetrahydroxy-5β(H)-cholest-7-en-6-one	[75]
	*Cucubalus baccifer*	2,22-dideoxy-20-hydroxyecdysone 3β-*O*-β-d-glucopyranoside	[76]
	*Sagina japonica*	japonicone	[77]
Commelinaceae	*Cyanotis achnoidea*	11α-hydroxyrubrosterone	[35]
		dacryhainansterone	
		calonysterone	
		cyanosterone A	
		cyanosterone B	
		22-oxo-ajugasterone C	
		22-oxo-20-hydroxyecdysone	
		ajugasterone C 2-acetate	
		shidasterone3-acetate	
		3β,4α,14α,20*R*,22*R*,25-hexahydroxy-5α-cholest-7-en-6-one	
	*Cyanotis longifolia*	5β-hydroxypoststerone	[31]
		14,15-dehydro-poststerone 2-acetate	
		poststerone 2-acetate	
		24-epi-atrotosterone A	
		ajugasterone C 3-acetate	
	*Callisia fragrans*	callecdysterol A	[78]
		callecdysterol B	
		callecdysterol C	
Dioscoreaceae	*Dioscorea dumetorum*	(20*R*)-5β-11α,20-trihydroxyecdysone	[79]
Gleicheniaceae	*Diplopterygium rufopilosum*	(22*R*,24*R*,25*S*,26*S*)-2β,3β,14α,20*R*-tetrahydroxy-26α-methoxy-6-oxo-stigmast-7-ene-22,26-lactone	[80]
		(22*R*,24*R*,25*S*)-2β,3β,14α,20*R*,26*S*-pentahydroxy-6-oxo-stigmast-7-ene-22,26-lactone	
		(22*R*,25*S*)-2β,3β,14α,20*R*,24*S*-pentahydroxy-6,26-dioxo-stigmast-7-ene-22,26-lactone	
Lamiaceae	*Ajuga taiwanensis*	ajugalide-E	[81]
	*Ajuga macrosperma*	breviflorasterone	[82]
		ajugacetalsterone C	
		ajugacetalsterone D	
	*Ajuga decumbens*	decumbesterone A	[83]
		ajugacetalsterone E	
	*Ajuga nipponensis*	22-dehydrocyasterone-2-glucoside	[84]
		ajugacetalsterone A	
		ajugacetalsterone B	
	*Ajuga turkestanica*	25-hydroxy-atrotosterone A	[85]
		11-hydroxy-cyasterone	[86]
		11-hydroxy-sidisterone	[87]
		turkesterone 22-acetate	
		22-oxo-turkesterone	
		11-hydroxy-Δ^24^-capitasterone	
		turkesterone 20,22-acetonide	
	*Ajuga reptans*	reptanslactone A	[88]
		reptanslactone B	
		sendreisterone	
	*Eriophyton wallchii*	28-epi-cyasterone	[89]
	*Vitex doniana*	21-hydroxyshidasterone	[90]
		11β-hydroxy-20-deoxyshidasterone	
		2,3-acetonide-24-hydroxyecdysone	
	*Vitex scabra*	24-epi-pinnatasterone	[91]
		scabrasterone	
	*Vitex cymosa*	26-hydroxypinnatasterone	[92]
	*Vitex canescens*	(24*R*)-11α,20,24-trihydroxyecdysone	
		11α,20,26-trihydroxyecdysone	
		24-methylshidasterone	
Liliaceae	*Asparagus filicinus*	stachysterone A 20,22-acetonide	[93]
Limnanthaceae	*Limnanthes alba*	limnantheoside C	[94]
Lygodiaceae	*Lygodium japonicum*	lygodiumsteroside A	[95]
Malvaceae	*Sida rhombifolia*	25-acetoxy-20-hydroxyecdysone-3-*O*-β-d-glucopyranoside	[96]
		pterosterone-3-*O*-β-d-glucopyranoside	
		ecdysone-3-*O*-β-d-glucopyranoside	
	*Sida spinosa*	20-hydroxy-24-hydroxymethyl ecdysone	[97]
	*Sida glutinosa*	glutinosterone	[98]
Menispermaceae	*Sphenocentrum jollyanum*	sphenocentroside A	[99]
		sphenocentroside B	
	*Cyclea barbata*	cycleasterone A	[100]
	*Diploclisia glaucescens*	3-deoxy-1β,20-dihydroxyecdysone	[101]
		2-deoxy-5β,20-dihydroxyecdysone	[102]
		diploclidine	
	*Fibraurea tinctoria*	fibraurecdyside A	[103]
Polypodiaceae	*Polypodium vulgare*	5-hydroxyecdysone	[104]
		20-deoxyshidasterone	
		polypodine B 2β-d-glucoside	
	*Microsorum scolopendria*	20-deoxymakisterone A	[105]
		25-epi-amarasterone A	
		25-deoxyecdysone 22-β-d-glucoside	
	*Microsorum membranifolium*	*E*-2-deoxy-20-hydroxyecdysone 3-caffeate	[106]
		2-deoxyecdysone 3-ferulate	[107]
		2-deoxyecdysone 25-α-l-rhamnopyranoside	[108]
	*Lepidogrammitis drymoglossoides*	ponasteroside B	[109]
Polyporaceae	*Polyporus umbellatus*	(20*S*,20*R*,24*R*)-16,22-epoxy-3β,14α,23β,25-tetrahydroxyergost-7-en-6-one	[110]
		(23*R*,24*R*,25*R*)-23,26-epoxy-3β,14α,20α,22α-tetrahydroxyergost-7-en-6-one	[111]
		polyporoid A	
		polyporoid B	
		polyporoid C	
Rhodomelaceae	*Laurencia alfredensis*	alfredensterol	[112]
		3-deacetoxy alfredensterol	
		14α-hydroxy alfredensterol	
Taxaceae	*Taxus cuspidate*	7,8β-dihydroponasterone A	[113]
	*Taxus canadensis*	ponasterone A 20,22-*p*-hydroxybenzylidene acetal	[114]
		ponasterone A 20,22-acetonide	

## Data Availability

Not applicable.

## Abbreviations

2,4-D	2,4-dichlorophenoxy acetic acid
20-HE	20-hydroxyecdysone
BR	brassinosteroid
CK	cytokinin
DMAPP	dimethylallyl diphosphate
EC	ecdysteroid
ET	ethylene
GA	gibberellin
HPLC	high-performance column chromatography
IPP	isopentenyl diphosphate
iPR	isopentenyladenosine
JA	jasmonic acid
MDA	malondialdehyde
MeJA	methyl jasmonate
MeOH	methanol
MEP	2C-methyl-erythritol-4-phosphate
MVA	mevalonic acid
NaOH	sodium hydroxide
NMR	nuclear magnetic resonance
PE	phytoecdysteroid
PIDA	phenyliodine(III) diacetate
PIFA	iodobenzene I,I-bis(trifluoroacetate)
ROS	reactive oxygen species
RuBP	ribulose 1,5-bisphosphate
SOD	superoxide dismutase
